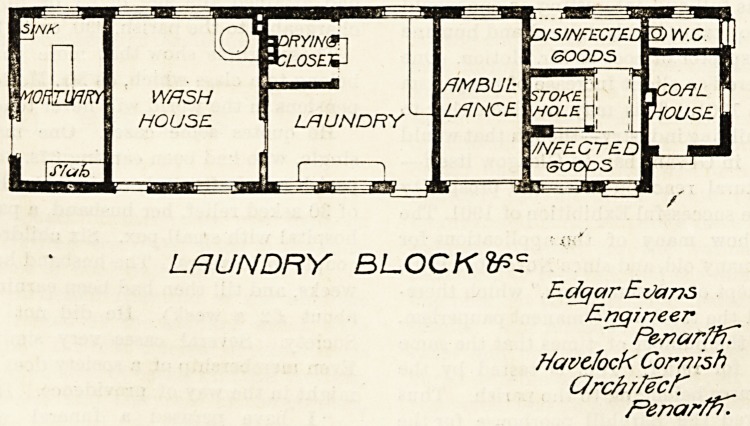# Penarth Hospital for Infectious Diseases

**Published:** 1903-04-25

**Authors:** 


### PENARTH HOSPITAL FOR INFECTIOUS DISEASES.

This hospital, which is now being built, stands on a plot of land at Llandough, near Cardiff, and it is intended for the Penarth United Sanitary District, the population of which is about 13,000. It would seem that the district is an unusually healthy one, for it had the lowest death-rate in the County of Glamorgan for 1901, being only 12·2 per 1,000. Hitherto a temporary isolation hospital has been in use, and up to the date of our information 323 cases of diphtheria had been dealt with therein with a mortality of 13. Of four cases of enteric fever two were imported. The drains throughout the district have been overhauled, large flushing tanks provided, and tall ventilating shafts put up.

The site of the hospital is nearly three and a half acres in extent, and the surface varies from 130 feet to 195 feet above the ordnance datum. It slopes towards the south. The soil is hard red marl. The hospital can be easily reached from all parts of the Penarth district. The whole institution consists of four blocks, namely, a block for 12 patients, an isolation block for four patients, a laundry block, and an administration block. The three last-named blocks are almost in line, and are placed near the southern boundary of the plot of land; therefore, the block for 12 beds is to the north, and space for another fever block has been left to the east of the one now erected.

In general plan this fever block very closely resembles that published by the Local Government Board, of which it can hardly be said that it represents any high flight of official architectural genius; but at least it provides a reasonable amount of cubic space per patient and fair cross ventilation, so it may pass muster. Near the entrance of this block are a discharging-room and a bath-room, so arranged that a patient on discharge can straightaway leave the hospital after being bathed and dressed. The isolation block for four beds may have applied to it the same remarks as the fever block. The fever block is warmed by stoves in the centre of the wards, and the isolation block by open fireplaces in the sides of the wards.

The laundry block is conveniently enough arranged. It contains a mortuary at one end, and an ambulance-room is placed between the laundry proper and the disinfecting-room.

The administration block has nothing strikingly original

WARD BLOCK.

ISOLATION BLOCK.

BLOCK PLAN.

LAUNDRY BLOCK &c.

Edgar Evans

Engineer

Penarth.

Havelock Cornish

Architect.

Penarth.

about it, but it contains all the rooms usually considered necessary for a small hospital. The first floor of this block provides the bedrooms for the staff. The chief elevation faces south-west, and thus it commands all the other blocks—a point of importance.

The building is of red brick, and the walls are constructed with an air space of two inches, whereby dryness and warmth are ensured to a greater or lesser extent. Water is obtained from the Cardiff water mains, and gas from the Gaslight and Coke Company. The sewers will be connected with the system which discharges into the Ely tidal harbour. The total cost will be £7,800. This includes furniture, road-making, and other things usually considered as extras to the contractor's tender. The architects are Messrs. Edgar Evans and Mr. H. Cornish. Mr. D. G. Price is the contractor. While we have no doubt that this hospital will satisfactorily answer its intended purpose, we could wish that architects, when planning these district hospitals, would show more originality in the conception of them. Hospital architecture has by no means run through all its phases. There is much to be done before the summit is reached, and it will only be reached by a process of elimination on one hand and addition on another. By these means the fittest will be evolved, and the fittest will survive. That consummation should be hastened.

## Figures and Tables

**Figure f1:**
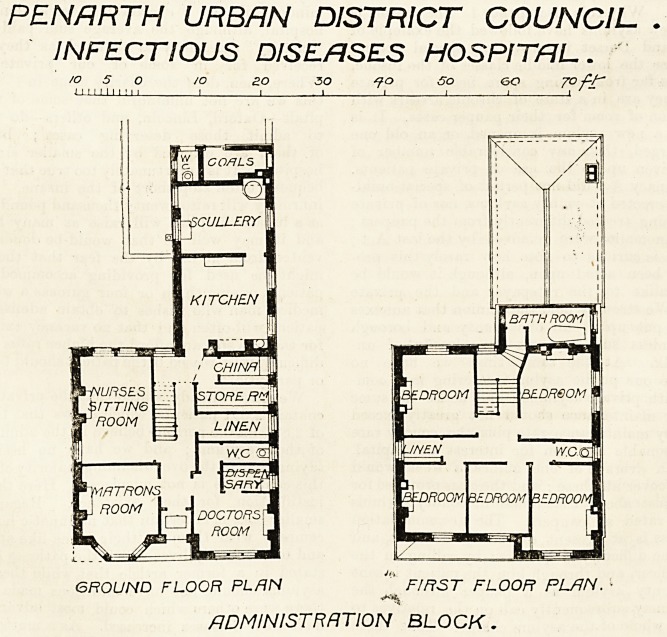


**Figure f2:**
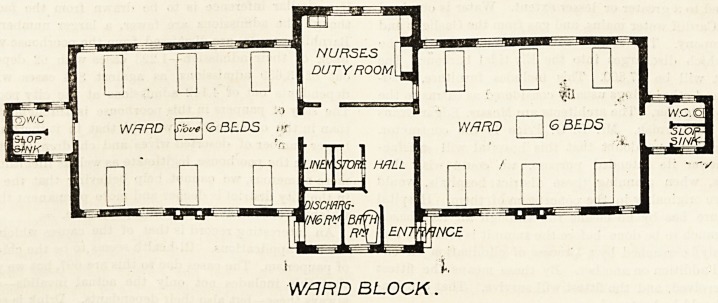


**Figure f3:**
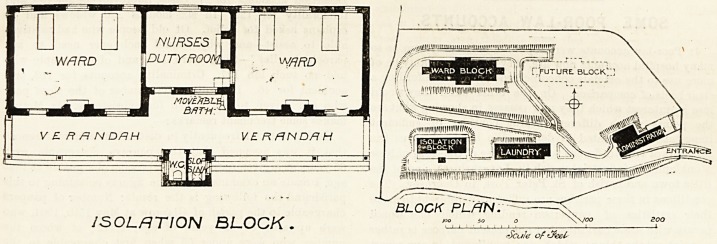


**Figure f4:**